# Measuring patient experiences in a Children’s hospital with a medical clowning intervention: a case-control study

**DOI:** 10.1186/s12913-020-05128-2

**Published:** 2020-04-26

**Authors:** Nina Karisalmi, Katja Mäenpää, Johanna Kaipio, Pekka Lahdenne

**Affiliations:** 1 Department of Computer Science, Aalto University, P.O. Box 15400, FI-00076 Aalto, Finland; 2 Hospital for Children and Adolescents, Helsinki University Hospital, Helsinki, Finland

**Keywords:** Patient experience, children’s hospital, Children, Parents, Digital survey tool, Case-control study, Medical clowns

## Abstract

**Background:**

Because the healthcare sector is shifting to a customer-oriented approach, it is important to understand experiences of children as users of healthcare services. So far, studies that measure the influence of medical clowning on patient experiences are scarce. This study aims to measure experiences of children and their parents during day-surgery in hospital setting.

**Methods:**

A case-control study was conducted in a large Finnish children’s hospital. Seventy children aged 4–17 years coming for a minor operative procedure including pre-operative cannula insertion prior to surgery were included. Thirty-eight children were exposed to the medical clowning intervention and 32 children (the reference group) did not receive exposure to medical clowning. A novel digital survey tool was used to measure patient experiences before and after the insertion of a venous cannula needed for anaesthesia. The children were asked about their emotions, anxiety levels, the pain from the cannula insertion and the best and worst things about the hospital. The parents were asked about their emotions, expectations and the fluency of the procedure and the hospital day.

**Results:**

Before the procedure, 32% or 36% of the children in the intervention group and 44% or 28% of those in the reference group expressed positive or neutral emotions, respectively. After the procedure, 76% or 63% of children in the intervention group or reference group, respectively, expressed positive emotions. The intervention group rated the medical clowns as the best aspect of the hospital day. Both groups reported that the best aspects of the hospital day were related to the nurses and food and the worst were related to waiting and pain. Most commonly the parents felt uncertainty, anxiety or calmness before the procedure and relief afterwards. Their expectations towards the procedure related to its success and the certainty of the diagnosis.

**Conclusions:**

The results show a trend towards more positive emotions in children with exposure to medical clowning. The digital survey tool was suitable for gathering information about the experiences of children and their parents. Information on emotions and expectations of children and parents during a procedure is useful when improving the quality of healthcare services.

**Trial registration:**

Current Controlled Trials NCT04312217, date of registration 17.03.2020.

Retrospectively registered.

## Background

Because the customer-oriented approach has recently become more prevalent in the healthcare sector, patient experience is now an important and well-recognised research topic. Healthcare organisations have acknowledged that patient experience significantly affects the perceived quality of care [1–3]. Examining elements which influence experiences of patients can be used to evaluate the effects of interventions and thereby improve the quality of healthcare services. However, studies measuring patient experience during an intervention are scarce.

### Patient experience and related concepts of human experience

By definition, an experience refers to “an event or occurrence which leaves an impression on someone” [4]. Likewise, an emotion is “a strong feeling deriving from one’s circumstances, mood, or relationships with others” [5]. Multidisciplinary studies of human experiences have resulted in different conceptualisations of the phenomena. For instance, the concepts of user experience, customer experience and quality-of-experience have been widely accepted and utilised in the fields of user-centred design and service engineering.

In the healthcare field, the relatively new concept of *patient experience (PX)* is often combined with related terms like patient perception, patient satisfaction and patient engagement [1–3, 6].

The most commonly cited definition for PX is as follows [7]: “The sum of all interactions, shaped by an organization’s culture that influence patient perceptions across the continuum of care”. Because these interactions and events are interconnected, they cannot be analysed in isolation [8]. Based on a literature review, another description of PX emphasises the following central themes: PX is more than satisfaction alone, continuum of care, focus on expectations, individualised care and alignment with patient-centred care principles [9].

### Measuring PX

Experiences can be investigated with both qualitative and quantitative methods. Because qualitative methods for examining PX (i.e. diaries, in-depth interviews and focus groups) provide participants with the flexibility to use their own words and descriptions, they can result in deeper insights into the phenomena [1, 10, 11]. Several researchers have applied qualitative methods to study the experiences of hospitalised children [12–16]. These studies have highlighted the children’s fears and worries, the importance of communication, their preference for care at home, as well as differences in categories of their experienced pain and the significance of entertainment. In contrast, quantitative methods, such as surveys and questionnaires [1, 10], generally use predetermined questions and larger sample sizes [1], and they may generate data for making comparisons, identifying patterns and monitoring changes [1, 10, 11]. One widely known tool is the Hospital Consumer Assessment of Healthcare Providers and Systems (HCAHPS) survey [17].

### Medical clowning

Medical clowns are professional performers [18] whose goal is to support the child during critical times of care, such as the transition to a procedure or cannula insertion [19]. Medical clowns are recognised members of the medical team, and they work in close collaboration with other healthcare professionals, such as nurses and anaesthesiologists. By providing support and keeping the child calm, medical clowns help the other professionals to focus on providing care and treatment [19]. Medical clowns’ methods help children deal with challenging emotions — such as sadness, stress, helplessness and even fear — and include play, music, magic, pantomime and soap bubbles [18, 20–22].

The medical clowning field began in 1980 in the United States and Canada, and it has now spread to dozens of countries [22, 23]. Previous studies on medical clowning have suggested that children and parents who had a medical clown present during their procedures were less anxious than those who did not [24–26]. In addition, the presence of a clown has reportedly been therapeutic and empowering for both children and their parents [27]. Significantly, it can positively change the children’s perceptions of the hospital and their experiences of the examination [28].

The context of the empirical study is medical clowning in the children’s hospital. The study was conducted in the Helsinki Children’s Hospital, which has collaborated with the Finnish Medical clown association since 2002. Medical clowns have the same hygiene and confidentiality rules as other healthcare professionals in the hospital [18].

### Aim of the study

The aim of this study was to measure experiences of children and their parents during day-surgery in hospital setting. For the purposes of our study, PX was described to include all interactions that could influence patient perceptions of healthcare services, including emotions, anxiety levels and the best and worst aspects of the hospital stay. Based on earlier studies on the influence of medical clowning on PX [18, 25, 26, 29], we hypothesised that medical clowns would have a positive impact on the children during their hospitalisation.

The research questions were as follows:

- *RQ1: How does engagement with a medical clown during pre-operative cannulation impact on children and their parents’ PX?*
- *RQ2: How feasible is a novel data collection tool for measuring PX and the impact of an intervention on the PX of families?*

The empirical study was part of the Lapsus research project [30], which has received permission from the Helsinki University Hospital’s ethics committee.

## Methods

The case-control study was designed to measure the experiences of children and their parents during a minor operative procedure including pre-operative cannulation prior to surgery at a Finnish children’s hospital. Medical clowning was chosen as the intervention. While the patients in the reference group did not have a medical clown present, their day-surgery process was otherwise routine.

### The digital survey tool

For the study, a novel digital survey tool was developed to measure the impact of an intervention on children’s and their parents’ experiences. The experiences were measured before and after the operation. The measurement focused on emotions, experiences of pain and perceptions of the best and worst aspects of the hospital stay. The Plutchik’s wheel of emotions [31] was used as a source of inspiration for choosing the emotions for the survey. Three focus areas were selected for the following reasons. First, we hypothesised that the children would feel anxious before a procedure [12] and that medical clowning would likely influence this emotional response [26]. Second, we anticipated that the children would dislike the cannula insertion due to its invasiveness [12–15]. Third, we took into account the results of earlier studies and expected that the children’s experiences at the hospital would not be exclusively negative [12].

One aspect that seems to affect the outcomes of PX measurements is timing [1]. The more time between the clinical encounter and the PX measurement, the worse the reported experiences [32]. Thus, to obtain accurate results, a PX measurement should occur as close to the encounter as possible. In this study, the children and their parents were asked the first set of questions once they had entered the Day Surgery Unit and given their consent to participate in the study. Before the procedure, the patients were asked about their current emotion (Q1) and their anxiety level (Q2) (see Table 1). Once the procedure was completed and the children fully recovered from the anaesthesia, the children were asked about their current emotion (Q1), their anxiety level (Q2), how much the cannula insertion hurt (Q3) and what the best and worst things were for them about the hospital day (Q4 and Q5). In addition, because of their cognitive abilities, the older children from 11 to 17 years of age were given an open feedback question (Q6) to elaborate more on their experiences of that hospital day in general or related to the procedure and the medical clown.

**Table 1:**
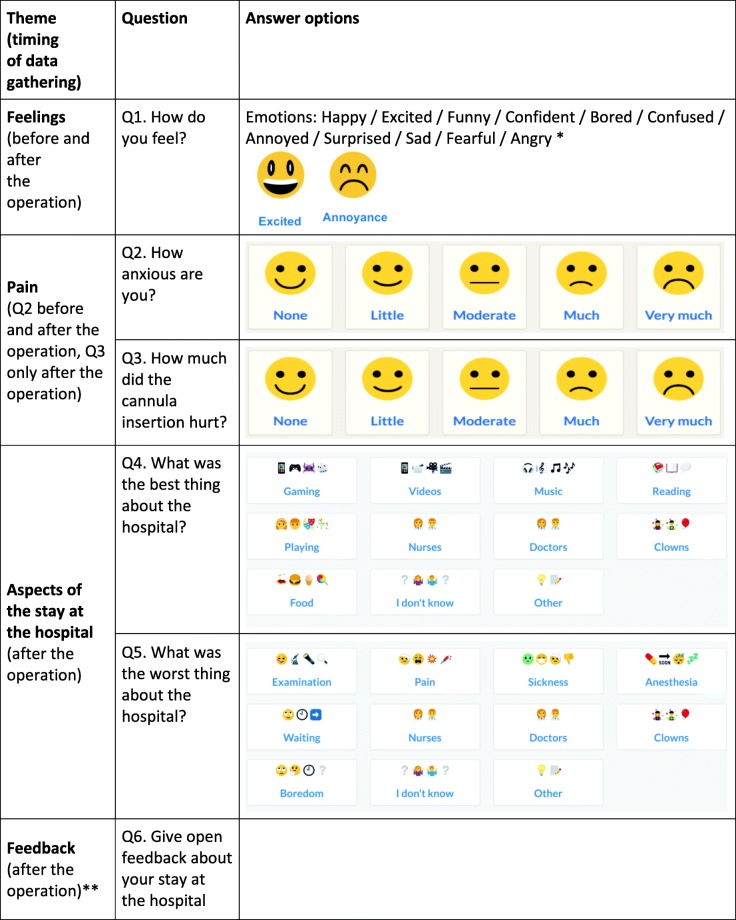
Questions and response options in the survey of children with the timing of data gathering included.

* Emotions Flash Cards [33] were used as an inspiration. For the table, we have presented only two of the eleven emojis with their corresponding emotions due to copyright issues

** Q6 was presented only to children over 11 years of age, and it was optional. Q1-Q5 were compulsory for all children

Surveys can use visuals, such as symbols, faces or emojis, to make them more suitable for illiterate children to answer. Face scales are used by many hospitals to measure the pain or anxiety of children. These scales have been suitable for children as young as 4 years old [34, 35]. Additionally, emotion cards have been used in the hospital context to investigate experiences of children [36]. Since the digital survey tool was designed to be understandable to children as young as 4 years of age, we used emojis for informative and unambiguous visuals (see Table 1). The age limit was set to 4 years of age based on the findings from our pilot testing, which indicated that 3-year-old children answer inconsistently to repeated questions.

The digital survey tool was pilot tested on 2 days with nine children (four from the intervention group, five from the reference group). Based on the findings of the pilot, the frequency of the data gathering during the procedure was reduced from three to two sessions. The initial plan was to ask the patients different questions twice after the procedure. However, since this was found to be time-consuming and repetitive, the two initial survey sets were merged together.

The first question of the survey (Q1) was presented with 11 different emoji faces and correspondently the second (Q2) and the third (Q3) questions with five faces (see Table 1). Of these, the child could only select one per question. Similarly, visual answer options were used for the questions on the best and worst things about the hospital (Q4-Q5) (Table 1). The original questions were in Finnish and have been translated into English for this article.

The parents answered questionnaires before and after the procedure while their children answered the survey. Parents provided their background information and answered questions related to their emotions, their expectations and the fluency of the procedure and the hospital day (see Table 2).

**Table 2:** The questions answered by the parents before and after the procedure

Questions for the parents before the procedure	
Background information	• Child study number ^a^
	• Relationship with the child: Mother / Father / Other
	• Does the child have a diagnosis yet? Yes / No
	• If the child has a diagnosis already, is the disease a: Short term disease / Long term disease / No diagnosis yet
	• Have you visited this children’s hospital before? Yes / No
	• Are you familiar with the child’s upcoming procedure? Yes / No
	• Has your child undergone the upcoming procedure before? Yes / No
Emotions and expectations	• How is your child feeling about the procedure in your opinion? E.g. happy, relieved, nervous, frustrated … ^a^
	• How are you feeling about the upcoming procedure? ^a^
	• What expectations do you have for the procedure? ^a^
	• On a scale from 1 to 5, are you currently feeling uncertainty, fear or nervousness towards your child’s sickness or disease? ^b^
**Questions for the parents after the procedure**	
Background information	• Child study number ^a^
	• Relationship with the child: Mother / Father / Other
Emotions, fluency of the procedure and fluency of the hospital day	• On a scale from 1 to 5, how well did the procedure go as a whole from your point of view?^c^
	• On a scale from 1 to 5, how well did the procedure meet your expectations? ^c^
	• What is your topmost feeling at the moment? ^a^
	• On a scale from 1 to 5, are you currently feeling uncertainty, fear or nervousness towards your child’s sickness or disease? ^b^
	• Open feedback on the study or the hospital day ^a^

^a^Open answer

^b^Likert-scale from 1 (strongly agree) to 5 (strongly disagree)

^c^Likert-scale from 1 (very poorly) to 5 (excellently)

### Implementation of the digital survey

The digital survey was presented to the children on a tablet, and it was implemented with Django and React. During the data gathering, the survey was hosted on the cloud application platform Heroku to enable its simultaneous usage by the patients. The emojis used in the survey were provided by EmojiOne [37]. The questions for the parents were implemented with Google Form.

### Procedure

**Fig. 1:**
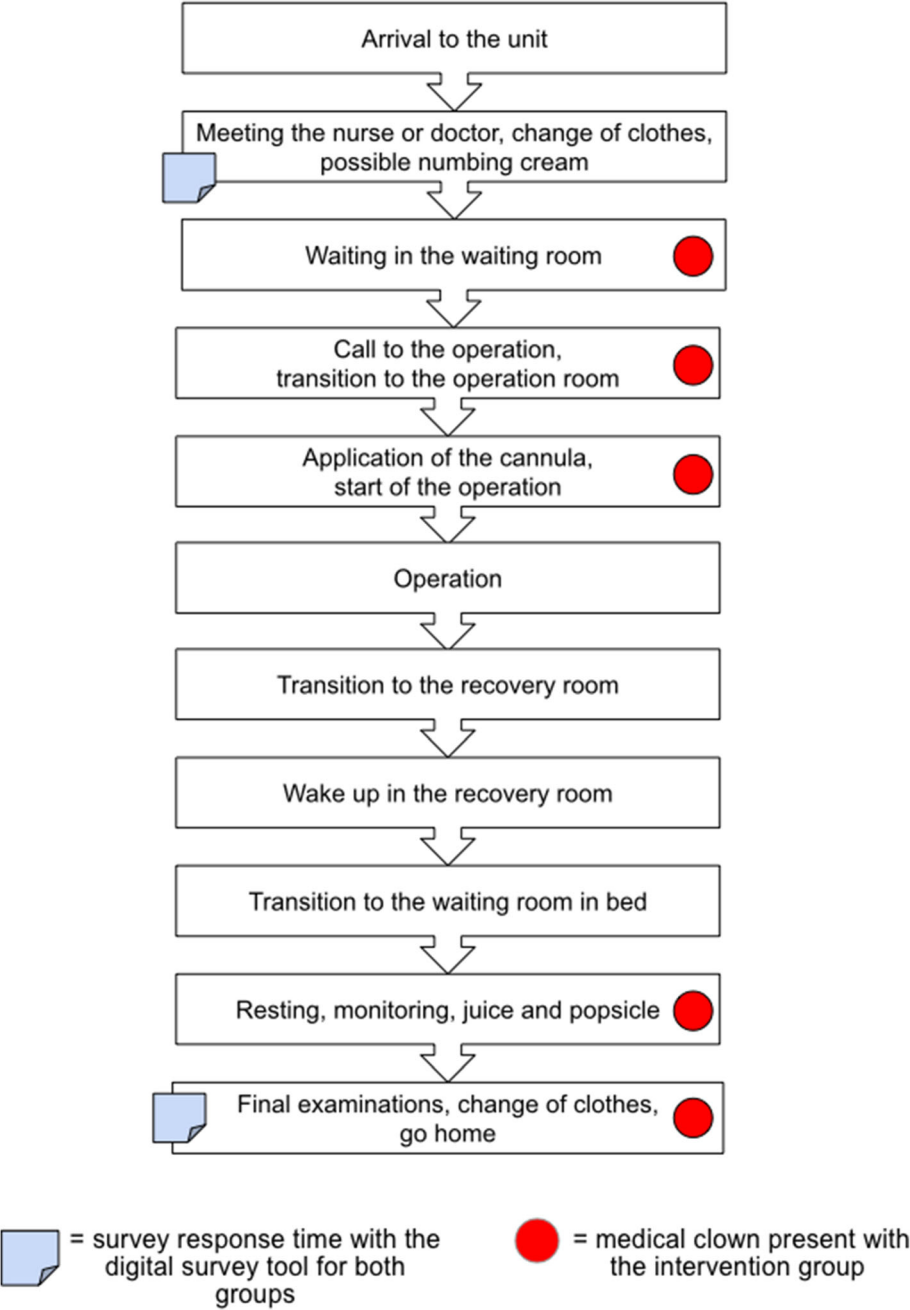
Illustration of the journey of children during a day-surgery operation with and without a medical clown, featuring the points of survey responses

The points of measurement were defined based on the patient journey map (Fig. 1), which illustrates the detailed steps of the procedure. In particular, it covers the insertion of the venous cannula, the anaesthesia at the unit, the patient’s actions and the different actors at the hospital. Moreover, it indicates the steps where the medical clown is present. The map was created in collaboration with children’s hospital personnel, as it was based on interviews with the personnel and observations of medical clowns and their work [38].

During May and June 2018, the data were collected for a period of 6 weeks in the Day Surgery Unit of the Helsinki Children’s Hospital. The study weeks were arranged in a similar manner, including two reference days without the preoperative medical clown and two days with the preoperative medical clown at the unit. Except the presence or absence of the medical clown, the procedure remained the same during the study days. Hence, the family was assigned to the group depending on the weekday they arrived to the hospital: two days in the week were intervention days with the medical clown present and two days reference days with no medical clowns present at the unit. If they fulfilled the inclusion criteria for the study, all patients and their parents coming to the Day Surgery Unit for a procedure were asked to participate in the study. The inclusion criteria were for participants to understand the Finnish language, the child to be between 4 and 17 years of age and their procedure to include the insertion of a cannula. Written consent was requested from the child and the accompanying guardian. Each participant was provided with a special number, which was used throughout the study instead of a name or other personal information. No personal data of the participants were collected. The only information that the patients provided to the digital survey tool was their age.

The study had the assistance of a dedicated research nurse who was familiar with the practices of the unit. The research nurse communicated with the children and the parents throughout the study. For the youngest participants, the research nurse read the questions out loud, but she otherwise did not influence the participants or their responses.

A short survey as a paper version was also included for the operating theatre nurses, who answered to it directly after the procedure [38]. Their questions were related to the success of the cannulation, their estimation of the child’s anxiety, the presence of the clown and administration of premedication. This paper focuses on the experiences of the children and their families and therefore the results of the nurses are not analysed in this paper.

### Data and analysis

Of the 103 children and their parents, 98 (95%) consented to participate in the study. Of the 98 patients, 70 received a cannula for their procedure and were thus included in the analysis. Participants who had a local anaesthesia or mask anaesthesia were excluded.

The research data were obtained with the digital survey tool in a JavaScript Object Notation (JSON) format, but they were later converted to a comma-separated values (CSV) format to enable their analysis with Excel. The data comprised of each participant’s research number, question set and answers. The data were analysed by the researchers in Excel. Before the analysis, the data were grouped into the intervention and reference groups, both for the children and their parents. In our analysis of the children’s surveys, the results of question 1 (Q1, Table 1) were grouped into three thematic categories — positive, neutral and negative emotions — because this grouping enabled better comparisons than the use of eleven different emotional categories. For the purposes of this study, the five-point scale assessments in Q2 and Q3 had two categories combined. “Very much” (5) and “Much” (4) were combined to form the category “Much or very much”. Similarly, “None” (1) and “Little” (2) formed the new category “None or little”. The third category remained “Moderate”. Open feedback was gathered from children 11 years of age or older, since based on our earlier study with pediatric patients, children 10 years of age or younger may repeat what they have heard from their parents [39].

In the parents’ survey, only the question about the child’s study number was compulsory. Some of the parents’ questions (see Table 2) contained a numerical scale. For the purposes of this study, the five-point Likert scale assessments “Strongly agree” (1) and “Agree” (2) were combined to form the category “Agree”. Similarly, “Strongly disagree” (5) and “Disagree” (4) formed the new category “Disagree”. The third category remained “Not sure”. Further, Likert scale assessments “Very poorly” (1) and “Poorly” (2) were combined to form the category “Very poorly or poorly”, as well as “Excellently” (5) and “Well” (4) formed the new category “Well or excellently”. The third category remained “Fairly well”. Data from the open questions were grouped into themes following the content analysis method [40].

## Results

### Participant characteristics

Seventy children participated in the study, of which 38 belonged to the intervention group and 32 to the reference group (Table 3). Of the patients, 36 were boys and 34 were girls. The mean age was 8.5 years for the intervention group and 10.3 years for the reference group. Of the families included, 77% (*n* = 54) had prior experience visiting the children’s hospital for a procedure (Table 4).

**Table 3:** Demographics of the children

	Intervention group (***n*** = 38)	Reference group (***n*** = 32)	Total (n / %)
**Gender (n)**			
Male	17	19	36 / 51
Female	21	13	34 / 49
**Age in years (n)**			
4	3	5	8 / 11
5	5	4	9 / 13
6	4	1	5 / 7
7	5	1	6 / 9
8	2	2	4 / 6
9	8	3	11 / 16
10	2	1	3 / 4
11	2	1	3 / 4
12	2	2	4 / 6
13	2	0	2 / 3
14	2	2	4 / 6
15	1	4	5 / 7
16	0	5	5 / 7
17	0	1	1 / 1
Mean age	8.5	10.3

**Table 4:** Characteristics of the families based on the background information from the parents’ questionnaires

**What is your relationship to the patient? (n / %)**	
Mother	52 / 74
Father	17 / 24
Other	1 / 2
**Do you have prior experience of the children’s hospital? (n / %)**	
Yes	54 / 77
No	16 / 23
**Does your child have a diagnosis yet? (n / %)**	
Not yet	29 / 42
Diagnosed less than a year ago	17 / 24
Diagnosed over a year ago	24 / 24

### Experiences of children

Before the procedure, the patients in the intervention group experienced a mixture of positive (32%, *n* = 12), neutral (36%, *n* = 14) and negative emotions (32%, *n* = 12). After the procedure, 76% (*n* = 29) expressed positive emotions (Table 5). In the reference group, 44% (*n* = 14) of patients experienced positive emotions before the procedure and 63% (*n* = 20) after the procedure.

**Table 5:** The most common emotions of children before and after the procedure (Q1)

Category	Emotion (n, in brackets %)	Intervention group (***n*** = 38)		Reference group (***n*** = 32)	
		Before^**a**^	After^**b**^	Before^**a**^	After^**b**^
Positive	Joy	6 (16)	12 (32)	7 (22)	8 (25)
	Fun	3 (8)	8 (21)	2 (6)	2 (6)
	Content	3 (8)	5 (13)	4 (13)	6 (19)
	Enthusiasm	0	4 (11)	1 (3)	4 (13)
	Total	12 (32)	29 (76)	14 (44)	20 (63)
Neutral	Confusion	6 (16)	2 (5)	5 (16)	7 (22)
	Boredom	5 (13)	4 (11)	4 (13)	3 (9)
	Surprise	3 (8)	1 (3)	0	1 (3)
	Total	14 (36)	7 (19)	9 (28)	11 (34)
Negative	Annoyance	6 (16)	2 (5)	2 (6)	0
	Fear	5 (13)	0	6 (19)	1 (3)
	Anger	1 (3)	0	1 (3)	0
	Sadness	0	0	0	0
	Total	12 (32)	2 (5)	9 (28)	1 (3)

^a^The first survey questions were given to children when they had a meeting with the nurse or the doctor before entering the waiting room (see Fig. 1)

^b^Questions were asked after the child’s procedure and before the family went home

In the intervention group, the most common emotions among the patients before the procedure were joy (16%, *n* = 6), confusion (16%, *n* = 6) and annoyance (16%, *n* = 6). After the procedure, they were joy (32%, *n* = 12) and fun (21%, *n* = 8) (Table 5). In the reference group, the most common emotions before the procedure were joy (22%, *n* = 7), fear (19%, *n* = 6) and confusion (16%, *n* = 5). After the procedure, they were joy (25%, *n* = 8), confusion (22%, *n* = 7) and content (19%, *n* = 6).

The average anxiety of children before the procedure was 2.53 (STD 1.0) for the intervention group and 2.25 (STD 1.1) for the reference group. After the procedure, the values were 1.37 (STD 1.1) for the intervention group and 1.41 (STD 0.9) for the reference group (Table 6).

**Table 6:** The anxiety (Q2) expressed by the children on a scale of 1–5

How anxious are you? (n, in brackets %)	Intervention group (***n*** = 38)		Reference group (***n*** = 32)	
	Before*	After**	Before*	After**
None (1) or little (2)	18 (47)	36 (95)	22 (69)	28 (88)
Moderate (3)	16 (42)	1 (3)	6 (19)	3 (9)
Much (4) or very much (5)	4 (11)	1 (3)	4 (13)	1 (3)
**Average**	2.53	1.37	2.25	1.41
**STD**	1.0	1.1	1.1	0.9
**Change**	1.16		0.84	

* The first survey questions were given to children when they had a meeting with the nurse or the doctor before entering the waiting room (see Fig. 1)

** Questions were asked after the child’s procedure and before the family went home

On the question of pain caused by the cannula insertion (Q3), 68% (*n* = 26) of the patients in the intervention group and 78% (*n* = 25) in the reference group answered that the insertion of the cannula hurt a little or did not hurt at all (Table 7).

**Table 7:** Pain caused by the cannula insertion (Q3) expressed by the children on a scale of 1–5

How much did the cannula insertion hurt? (n, in brackets %)	Intervention group (***n*** = 38)	Reference group (***n*** = 32)
None or little (1–2)	26 (68)	25 (78)
Moderate (3)	8 (21)	3 (9)
Much or very much (4–5)	4 (11)	4 (13)
**Average**	2.0	1.8
**STD**	1.2	1.3

In both groups, waiting and pain were the worst aspects of the hospital day (Q4 and Q5) (Table 8). In the intervention group, the preoperative medical clown was most frequently reported to be the best aspect of the hospital day. The themes from the open feedback answers (Q6) given by patients (5/48) aligned with the responses given to Q4 and Q5. Namely, the nurses were kind and the medical clowns were funny. These children also reported negative experiences with long waiting times.

**Table 8:** The best and worst aspects of the hospital day (Q4 and Q5) expressed by the children

	Intervention group (n / %)	Reference group (n / %)
**Best aspects of the hospital day**	1. Clowns (20 / 53%)	1. Nurses (8 / 25%)
	2. Nurses (5 / 13%)	2. Food (6 / 19%)
	3. Food (4 / 11%)	3. Games (5 / 16%)
	4. Playing (3 / 8%)	4. Videos, reading, other (each 2 / 6%)
**Worst aspects of the hospital day**	1. Waiting (12 / 32%)	1. Waiting (12 / 38%)
	2. Pain (9 / 24%)	2. Pain (8 / 25%)
	3. Anaesthesia (4 / 11%)	3. Anaesthesia (4 / 13%)
	4. Examination (2 / 5%)	4. Other (3 / 9%)

### Experiences of the parents

Of the 70 parents, 68 responded to the questionnaire about their experiences. Before the procedure, many of the parents expressed uncertainty, fear or nervousness (39% in the intervention group and 43% in the reference group, Table 9).

**Table 9:** Parents’ responses to whether they have a feeling of uncertainty, fear or nervousness towards the child’s sickness or disease

Are you currently feeling uncertainty, fear or nervous towards your child’s sickness or disease?	Before procedure^**a**^		After procedure^**b**^	
	Intervention group (***n*** = 38)	Reference group (***n*** = 30)	Intervention group (***n*** = 38)	Reference group (***n*** = 30)
Agree or strongly agree	39%	43%	13%	13%
Not sure	32%	27%	21%	20%
Disagree or strongly disagree	29%	30%	66%	67%

^a^Questions were asked after the parents had entered the Day Surgery Unit and given their consent to participate in the study

^b^Questions were asked after the child’s procedure and before the family went home

The expectations before the procedure were related to the following topics (Table 10): success of the procedure (36% of all responses, *n* = 20), the additional knowledge and certainty of the diagnosis (32%, *n* = 18) and the relief for the child’s symptoms caused by the procedure, thus making everyday life easier (27%, *n* = 15). These three themes emerged as the central themes in both groups’ answers. Of the parents’ feelings about the upcoming procedure, identified on the wheel of emotions, two emotions stood out: anxiety (46% of all the responses, *n* = 31) and calmness and restfulness (45%, *n* = 30). The most commonly mentioned feelings after the procedure were relief (69% of all responses, *n* = 47), pleasure and happiness (24%, *n* = 16). These themes were the most commonly mentioned ones by both groups, with other emotions being mentioned sporadically (under 8% of respondents).

**Table 10:** Expectations of the parents towards the upcoming procedure and their feelings before and after the operation (n, in brackets %). The emotions listed were mentioned at least twice in the open feedback

	Intervention group	Reference group	Total
**Expectations towards the procedure**^**a**^	***n*** **= 31**	***n*** **= 24**	***n*** **= 55**
The procedure will go well	12 (39%)	8 (33%)	20 (36%)
Get explicit diagnosis, get more information, get certainty	7 (23%)	11 (46%)	18 (32%)
Relieve child’s symptoms, everyday life gets easier	10 (32%)	5 (21%)	15 (27%)
Get guidelines for the treatment	2 (7%)	1 (4%)	3 (5%)
Get positive news, nothing surprising is found	1 (3%)	2 (8%)	3 (5%)
No expectations	2 (7%)	0 (0%)	2 (4%)
**Feelings about the upcoming procedure**^**a**^	***n*** **= 37**	***n*** **= 30**	***n*** **= 67**
Anxious	16 (43%)	15 (50%)	31 (46%)
Calm and restful	17 (46%)	13 (43%)	30 (45%)
Feel good, hopeful, content	3 (8%)	2 (7%)	5 (7%)
Worried	2 (5%)	2 (7%)	4 (4%)
Uncertain	0 (0%)	3 (10%)	3 (4%)
Annoyed	3 (8%)	0 (0%)	3 (4%)
**Feelings after the procedure**^**b**^	***n*** **= 38**	***n*** **= 30**	***n*** **= 68**
Relief	24 (63%)	23 (77%)	47 (69%)
Pleased, happy	11 (28%)	5 (17%)	16 (24%)
Anxious	2 (5%)	2 (7%)	4 (6%)
Boredom	2 (5%)	1 (3%)	3 (4%)

^a^Questions were asked after the parents had entered the Day Surgery Unit and given their consent to participate in the study

^b^Questions were asked after the child’s procedure and before the family went home

Over 90% of the parents in both groups perceived that the procedure went well and met their expectations (Table 11).

**Table 11:** Parents’ responses to questions about the procedure as a whole and how well the procedure met their expectations

	Intervention group	Reference group
**How well did the procedure go as a whole?**	***n*** **= 37**	***n*** **= 30**
Very poorly or poorly	0%	0%
Fairly well	5%	7%
Well or excellently	95%	93%
**How well did the procedure meet your expectations?**	***n*** **= 38**	***n*** **= 30**
Very poorly or poorly	3%	0%
Fairly well	5%	7%
Well or excellently	92%	93%

The open feedback, given by 49 parents (of which 31 from the intervention group and 18 from the reference group) was related to the successful procedure and hospital visit, contained appreciation towards the personnel and expressed annoyance at having to wait for a long time. In the intervention group, the most common feedback (23/31) was gratefulness for the amusement that the medical clown provided.

## Discussion

In the present study, the impact of a medical clown on children and their parents’ experiences on cannulation prior to surgery was evaluated. Additionally, a digital survey tool to assess children’s PX was developed and tested on children undergoing a day-surgery procedure.

### PX and medical clowning as an intervention

The present study suggests that medical clowns may have a positive effect on the PX of children and their parents. Obviously, the clowns may add to the joy that the staff routinely provides for children. These findings are consistent with earlier studies on medical clowning, which have discovered that the presence of a medical clown during the hospital day decreases the anxiety of both the children and their parents [18, 25, 26, 29].

Over 90% of the parents felt that the procedure was successful and progressed as expected. In this respect, the results do not show remarkable differences between the intervention and the reference groups. However, in their open comments, the parents in the intervention group reported that the preoperational clown decreased both their own and their child’s anxiety. The clown gave the family something else to think about before the procedure, thus helping them to cope with the long waiting times:

*“The medical clown amused the child and relieved anxiety while we were waiting for the procedure. I think that we will remember the medical clown for a long time. The hospital day went well, and the care was really good.”*

*“The medical clown was a lovely surprise, and it was wonderful, reassuring and liberating to laugh in a situation like this.”* (Quotes translated from Finnish)

The study also featured a short survey for the nurses in the operating room. The survey showed that the presence of a medical clown did not have an impact on the fluency or timing of the cannula insertion [unpublished].

### Feelings and expectations of children and their parents

The multiple feelings and expectations of the children were not unexpected and did not essentially differ between the intervention and reference group. However, the presence of medical clowning was highly appreciated. Our results are compatible with those from previous studies: children enjoyed the nurses [12–14, 41], food gave pleasure [12], waiting was boring [13, 41] and painless, non-invasive procedures were preferred [12–15, 39].

The parents also felt different emotions before and after the procedure. Many reported feeling either anxious or calm and restful before and relieved after the procedure. The parents’ expectations were mostly related to prospects of successful course of the procedure and to getting more information and certainty about the diagnosis. To provide support for parents, hospitals should meet this need for information when trying to improve the quality of the services. Meeting this need would probably enhance the parents’ PX. Additionally, we suggest that more research is needed at the conceptual level to approach PX and its related concepts of experience to support empirical studies on PX and PX quality measures.

### Digital survey tool for measuring PX and its evaluation

The design of the contents for the digital survey tool utilised earlier research on subjects like children’s pain, anxiety measurements and emotions [26, 29, 34, 36, 42–45]. Many of the previous studies used observer-based anxiety scales [18, 25, 26], such as the Modified Yale Preoperative Anxiety Scale (mYPAS), but some used subjective reporting, such as the Faces Pain Scale and Visual Analogical Scales (VAS) [29]. We did not use a standardised survey because, to the best of our knowledge, no premade tool fit for both the purpose of the study and the circumstances at the hospital was available.

Compared to a paper format, a digital tool for data collection had some advantages: privacy settings that ensured no one other than the researchers could see the responses after submission, adaptation to the questioning time, a digital interface that enabled even youngest children to answer the survey utilizing emoji faces and other figures without the need to write, suitability to the hectic hospital environment and the availability of both instant data gathering and feedback. However, the design of the survey tool had the following limitations: the different cognitive development stages of children from 4 to 17 years old, the hectic working pace at the hospital and the implementation of a digital survey on a tablet device. Children aged 4 to 17 were included because children as young as 4 are able to answer self-reported faces scales [34, 35] and the age limit of the children’s hospital was 17.

In our study, both the children and their parents gave positive feedback about the survey tool (data not shown). They reported that the visuals were enjoyable and familiar from interactions with other emojis and that the visuals made the survey itself quick to answer on the tablet device. In addition, the children could answer the questions independently, with the exception of the youngest children who were not able to read yet. These children received some help from their parents or the research nurse but mainly answered independently. In the study, the dedicated research nurse had an important role inviting the families to participate and providing them with the digital surveys during the procedure.

### Limitations

Our study has some limitations. First, the number of participants is only adequate for a feasibility study and for piloting the measurement of PX. While the participants represented their sample groups well, our conclusions should be treated with caution. Further research is needed to make generalisations and to study statistical differences between the intervention and reference groups.

Second, although all patients who visited the Day Surgery Unit and met the inclusion criteria participated, the demographic differences between the intervention and reference groups could have affected the results. Although the patients were arbitrarily allocated to the groups depending on their hospital days, those in the intervention group were a bit younger. However, the medical clowns can have meaningful encounters with differently aged children and thus, it is not a strictly controlling factor. Another drawback could be the differing attendance of the medical clown in the intervention group as the clown was present during the cannula insertion for 29 of the 38 patients (data not shown).

Third, the children may have found the question about their anxiety levels (Q2) to be somewhat ambiguous, even though in the pilot tests the participants evaluated the digital survey tool as clear, easy to use and suitable for its intended use (data not shown). To make the question about anxiety levels easier for illiterate children to understand, emojis were included as well as text explaining the scale from “none” to” very much”. The “none” emoji was a smiling face, whereas the “very much” emoji was a sad face. However, it remains unclear if the children perceived it this way or interpreted as “smiling anxiousness” and “sad anxiousness”.

## Conclusions

The findings of the study indicate a trend towards more positive emotions in children when exposed to medical clowning in hospital. The novel digital survey tool was found to be suitable for gathering PX information from children as young as 4 years old and their parents. Emojis allowed young children to answer the questions. For the purposes of improving perceived quality of healthcare services, investigation of patient experiences provides valuable information about emotions and expectations of child patients as well as their parents.

## Data Availability

The datasets generated and analysed during the current study are not publicly available since they contain information that could compromise research participant privacy/consent. The authors declare that the data supporting the findings of this study are available within the article. The authors of this article have conducted the study and they have the right to use the data and materials for research purpose.

## Abbreviations

HCAHPS: Hospital Consumer Assessment of Healthcare Providers and Systems
PX: Patient experience
